# Histone deacetylase inhibitors sensitize glioblastoma models to temozolomide and reprogram immunosuppressive myeloid cells

**DOI:** 10.21203/rs.3.rs-6573675/v1

**Published:** 2025-05-14

**Authors:** Golnaz Asaadi Tehrani, Becca Kubick, Maksym Zarodniuk, Meenal Datta

**Affiliations:** University of Notre Dame; University of Notre Dame; University of Notre Dame; University of Notre Dame

**Keywords:** HDAC, valproic acid, suberoylanilide hydroamic acid, chemotherapy resistance, neurospheres, macrophages

## Abstract

Histone deacetylase inhibitors (HDACis) are promising anti-cancer agents but remain underexplored in glioblastoma (GBM). This study evaluated the effects of three HDACis—CAY10603, vorinostat (SAHA), and valproic acid (VPA)—on human GBM cell lines (U87, MGG8) with immortalized human astrocytes (IHAs) as healthy controls. HDACis were tested alone or in combination with temozolomide (TMZ), the standard chemotherapy for GBM, in both 2D (monolayer) and 3D (neurosphere) cultures. Additionally, co-culture of GBM cells with macrophages (M0, biochemically differentiated from THP-1 human monocytes) was used to examine the impact of HDACis on cancer-immune interactions. Results demonstrated that all three HDACis significantly reduced cell viability and synergistically enhanced the effect of TMZ. CAY10603 and SAHA induced early apoptosis and upregulated caspase 3 (CASP3) expression, whereas VPA primarily induced late apoptosis and necrosis in GBM cultures. VPA induced both G0/G1 and G2/M cell cycle arrest, while SAHA and CAY10603 only induced G2/M arrest. mRNA expression analysis following HDACi treatment in U87 neurospheres revealed that HDACis inhibited expression of markers for epithelial-to-mesenchymal transition (EMT), proliferation, and stemness pathways. In U87-M0 co-cultures, we observed significant upregulation of stemness markers and the pro-inflammatory cytokine TNF-α following CAY10603 and VPA treatments. In contrast, TMZ monotherapy upregulated the expression of the immunosuppressive cytokine TGF-β. These findings suggest that HDAC inhibition—including the novel small molecule CAY10603—sensitizes GBM to temozolomide and confers potent anti-tumor effects that combat GBM (e.g., reducing proliferation, EMT, stemness). Our *in vitro* findings—e.g., with 3D neurospheres that better mimic physiological tumor growth than 2D monolayers—warrant future *in vivo* testing of HDACis alone or in combination with chemotherapy.

## Introduction

The origin and progression of glioblastoma (GBM) is influenced by both genetic and epigenetic modifications^1–3^. Histone deacetylases (HDACs) are epigenetic regulators of gene expression that remove acetyl groups from histones, a modification often associated with gene repression^4,5^. HDAC inhibitors (HDACis) enable acetylation of both histone and non-histone proteins. They exert multiple anti-tumor effects, most notably inducing differentiation, apoptosis, cell cycle arrest, susceptibility to chemotherapy, and inhibition of migration and angiogenesis^6–9^. Over the past 15 years, numerous HDACis have been studied preclinically in neuro-oncology, and three HDACis (vorinostat, romidepsin (FK-228), and panobinostat (LBH589)) have been tested in clinical trials for patients with primary and recurrent GBM^10–12^. Unfortunately, these agents have not significantly improved survival in GBM patients. The underwhelming clinical outcomes of HDACis in GBM treatment can be attributed to several factors, including inadequate disease modeling in preclinical studies, poor/heterogeneous blood-tumor barrier (BTB) penetration, and limited pharmacokinetic profiling of these drugs within the central nervous system (CNS)^10,13,14^. Early clinical trials indicate that HDACis from several structural classes are well-tolerated and exhibit therapeutic activity against a variety of human malignancies^15–17^, but the potential pleiotropic molecular mechanisms of these drugs in GBM are still being uncovered. Elucidating HDACi molecular mechanisms in GBM is relevant for the development of more specific and potent therapeutic strategies for this devastating disease.

Here, we characterized the pharmacological response of human GBM cell lines to two pan-HDACis currently under testing in clinical trials against GBM (SAHA, VPA) and a selective inhibitor of HDAC6 (CAY10603). Our results indicate that all three drugs exhibit anti-proliferative effects compared to TMZ monotherapy (the standard chemotherapy for GBM). Notably, the novel HDAC6 inhibitor exhibited a significant synergistic effect with TMZ and downregulated cancer stemness pathways. We also found that HDACis suppress the growth of GBM lines and induces histone H3 on lysine 27 (H3K27) hyperacetylation, cell death through CASP3 expression, and G0/G1, G2/M cell cycle arrest. We further observed the ability of HDACis to inhibit stemness, proliferation and EMT in neurospheres derived from U87 cells. We found that THP-1-derived macrophages promote the stemness of U87 cells via HDAC6/TNF-α/SOX2 pathway. Interestingly, histone deacetylase 6 (HDAC6) inhibition, suppressed transforming growth factor beta (TGF-β)/SRY-BOX2 (SOX2) and EMT pathway through tumor necrosis factor alpha (TNF-α) activation, and polarized macrophages from pro- to anti-tumor phenotypes. The data presented in this study provides the groundwork for future *in vivo* studies, and rational design of combinational therapeutic strategies to further improve patient survival in GBM.

## Materials and Methods

### 2D and 3D cell culture assays and drug treatment protocols

U87 (ATCC, Manassas, VA, USA) and IHA cells (Creative Bioarray, CSC-C12025Z, Shirley, NY, USA) were cultured at 37 °C in a humidified atmosphere with 5% CO₂ following our prior methods^18^. U87 cells were maintained in DMEM (Dulbecco’s Modified Eagle Medium; Invitrogen, Carlsbad, CA, USA) supplemented with 10% fetal bovine serum (Gibco, Grand Island, NY, USA) and 1% Penicillin-Streptomycin (Invitrogen, Carlsbad, CA, USA). IHAs were cultured in SuperCult IHA Media (Creative Bioarray, Shirley, NY, USA) with 1% IHA growth supplement, 2% fetal bovine serum, and 1% Penicillin-Streptomycin (all from Creative Bioarray, Shirley, NY, USA). MGG8 cells were cultured in neurobasal media (Life Technologies) supplemented with 1× B27, 5 mg/ml heparin (Stemcell Technologies), 20 ng/ml EGF (PeproTech), and 20 ng/ml basic FGF (PeproTech), following our prior methods^19^. For 3D culture experiments, U87 cells (5–8th passage) were seeded at a density of 100,000 cells/ml in a defined serum-free medium, NeuroCult^™^ NS-A Basal medium (Human) with NeuroCult^™^ proliferation supplement, antibiotics (penicillin 100 IU/ml, streptomycin 100 μm/ml), recombinant human epidermal growth factor (rhEGF, 20 ng/ml; R & D Systems, Minneapolis, MN, USA), basic fibroblast growth factor (bFGF, 10 ng/ml, R & D Systems), and a heparin solution (2 μg/mL). The medium was replaced every 2 days. Cells were allowed time to aggregate into neurospheres, which were then imaged using a light microscope (Eclipse; Nikon Corporation, Tokyo, Japan). When cells reached 75–80% confluency, they were treated with an HDACi (SAHA, CAY10603, or VPA) and/or TMZ for 24 – 48 hours. Following incubation, the cells underwent protein extraction and western blot analysis. Cell viability assays and fluorescence staining were also performed following the same treatment procedures.

### MTT assays for cell viability

U87 and IHA cells were plated at a density of 5 × 10^3^ cells/well in 96-well plates and incubated at 37 °C under 95% air and 5% CO_2_ for 24 hours. When the cells reached 75–80% confluency, they were treated for 24 or 48 hours with different concentrations of the drugs (SAHA: 0.5, 1, 2.0, 5.0, or 10.0 μM; CAY10603: 0.1, 1.0, 2.0, 5.0, or 10.0 μM; and VPA: 0.01, 0.1, 1.0, 4.0, or 10.0 mM). After incubation, the viability of the cells was assessed by MTT assay (Abcam, Waltham, MA, USA) according to the manufacturer’s protocol. After 24 or 48 hours of treatment, 50 μL of serum-free media and 50 μL of the MTT reagent were added to each well. The cells were incubated at 37 °C for an additional 3 hours. At the end of the specified incubation period, 100 μL of MTT solvent was added to each well. To solubilize the MTT-formazan precipitate, the plate was gently rotated on an orbital shaker for 15 minutes. The final absorbance was measured at 570 nm using a microplate reader. The data are expressed as the mean ± standard deviation. Experiments were performed in triplicate. The relative cell growth was calculated and expressed as IC50 values using GraphPad Prism 8.0 (GraphPad Software, Inc.). Untreated cells were chosen as a control, which were cells incubated with DMSO (solvent for TMZ) and DMSO (solvent for HDACis). The effect of the combination treatment of TMZ (50 and 100 μM) with HDACis was defined as a synergistic effect if CI < 1, an additive effect if CI = 1 or an antagonistic effect if CI >1.

### Flow cytometry studies for cell proliferation and apoptosis

Cell proliferation was assessed using propidium iodide (PI) staining, as PI binding to DNA is proportional to DNA content, allowing determination of cell cycle stage in each cell (Cell Cycle Assay Kit, Elascience). Cells were initially seeded in 6-well plates at a density of 1×10⁶ cells/well and incubated overnight in complete medium. Following PBS washing, cells were treated with drugs for 48 hours in complete medium. Cells were then fixed with 70% ethanol (18 hours, 4°C) and stained with 500 μL of PI solution (containing RNase) in the dark for 20 minutes at room temperature before flow cytometry analysis. Each experiment was performed in triplicate using a BD FACS Melody Cell Sorter (USA). For the apoptosis assay, U87 cells were seeded in 6-well plates at a density of 1×10^5^ cells/well and treated with an HDACi, with an untreated control included. After 48 hours of incubation, cell death was assessed by evaluating the loss of membrane integrity (high PI fluorescence) after treatment with PI solution. Phosphatidylserine externalization was measured using an Annexin V-FITC/PI double-staining kit (Cell Event, Invitrogen, USA), followed by flow cytometry analysis. Staining analysis categorized cells into four groups: viable (Annexin V− PI−), early apoptotic (Annexin V+ PI−), late apoptotic (Annexin V+ PI+), and necrotic (Annexin V− PI+).

### Protein preparation and western blot analysis

After 28 hours of drug treatment, the cells were lysed with radio-immunoprecipitation assay (RIPA) buffer containing a protease inhibitor cocktail (Sigma-Aldrich) and 17.4 μg/ml phenylmethylsulfonyl fluoride (Sigma-Aldrich) for 30 min at 4 °C. Cell lysates were centrifuged at 4 °C for 20 min at 14,000 rpm to clarify the samples from unbroken cells and organelles. The concentrations of proteins in the clarified samples were determined using the bicinchoninic acid (BCA) protein assay method (Thermo Fisher Scientific, Grand Island, NY, USA). The protein samples were then analyzed by western blot using 7.5–12% sodium dodecyl sulfate-polyacrylamide gel electrophoresis (SDS-PAGE). Equal concentrations of protein were loaded into each well, which was verified later by measurement of GAPDH levels. Following electrophoresis, proteins were transferred to nitrocellulose membranes, which were blocked using 5% nonfat dry milk and then probed with specific antibodies: HDAC1 (Ab109411), HDAC6 (Ab133493), H3K27ac (4729), Cas3 (Ab13847), and GAPDH (AM4300). Detection of specific protein bands on the membranes was achieved by incubating the membranes in a solution containing LumiGLO Reserve chemiluminescent substrate (KPL, Milford, MA, USA). Densitometric analyses were performed using the ImageJ program (National Institutes of Health, Bethesda, MD, USA).

### Fluorescent imaging of 2D/3D cultures for cell viability

The fluorescent caspase substrate DEVD-amc is a cell-permeant substrate specific for caspase-3/7 activity. It consists of a four-amino acid peptide (DEVD) conjugated to a nucleic acid-binding dye, amc (7-amino-4-methylcoumarin). The peptide sequence is based on the PARP cleavage site Asp216 for caspase-3/7. Un cleaved DEVD-amc is intrinsically nonfluorescent. During apoptosis, caspase-3 and caspase-7 enzymes are activated, leading to cleavage of the DEVD-amc conjugate. The liberated dye can bind to DNA, generating bright green fluorescence which can be measured at 535 nm. To determine the effects of drug treatment on apoptotic pathways, cells in 2D culture were treated with SAHA, CAY10603, or VPA for 48 hours. After drug treatment, the cells were washed and incubated with the caspase-3/7 green DEVD-amc substrate, DAPI, and PI for 15–30 minutes. Following staining, any resultant fluorescence in apoptotic cells was observed by fluorescence microscopy. Fluorescent staining was also performed on neurospheres derived from U87 cells following HDACi treatment. Cells were stained with calcein AM and PI, and then imaged using a Keyence BZ-X810 widefield microscope (Keyence, Itasca, IL, USA).

### Real-time quantitative PCR analysis of neutrospheres

Following 10 days of culture, spheres were quantified and then treated with an HDACi or TMZ for 48 hours. Total RNA was extracted using the Qiagen RNeasy Mini kit (Qiangen, Valencia, CA, USA) following the manufacturer’s instructions. The purity and quantity of all RNA samples were examined by NanoDrop (Thermo Fisher Scientific, USA). Gene expression was quantified by real-time quantitative PCR (RT-qPCR) using 100 ng of total RNA per reaction. TaqMan^®^ probes (Thermo Fisher Scientific) used for evaluating gene expression are the following: CTNNB1 (Hs00355045_m1), SOX2 (Hs04234836_s1), TRPM7 (Hs00559080_m1), ITGA5 (Hs01547673_m1), SNAIL2 (Hs00161904_m1), ZIC1 (Hs00602749_m1). The expression ratio was calculated by the ΔΔCt method as described by Livak and Schmittgen^20^, using GAPDH as the housekeeping gene for data normalization. Each sample was analyzed in triplicate.

### U87/THP1 co-culture and analysis of stemness and polarization markers

The co-culture system was assembled using a 6-well plate insert (0.4-μm porous; Corning, NY, USA) to create a transwell. THP-1 monocytes (1×10^6^ cells/mL) were seeded into these inserts and treated with 100 ng/mL PMA for 72 hours to stimulate differentiation into macrophages. The cells were then washed with phosphate-buffered saline (PBS) three times and cultured for 24 hours to exclude the interference of PMA. U87 cells (1×10^6^ cells/mL) were seeded in 6-well plates and incubated for 24 hours to allow for attachment. The inserts containing the THP-1-derived macrophages were placed directly on top of the wells containing the U87 cells. The cells of the resulting co-culture system were incubated in DMEM medium for 24 hours. U87 cells and THP-1-derived macrophages were also incubated individually in DMEM medium for 24 hours as the corresponding controls. Following the incubation, expression profiles of *SOX2* (Hs04234836_s1), *TGF-β* (Hs07289533_m1), and *TNF-α* (Hs00174128_m1) were analyzed by RT-qPCR.

### Statistical Analysis

The statistical analyses were performed using Student’s t tests or multiple t-tests with Bonferroni correction for multiple comparisons using Prism (GraphPad Software, Inc.). A confidence level of 95% was adopted and statistical differences between the means were considered significant at *p* < 0.05. Bar plots display the mean ± standard error of the mean (SEM) from independent measurements.

## Results

### HDACis reduce GBM cell viability as monotherapies and synergistically in combination with TMZ

**Figure 1:**
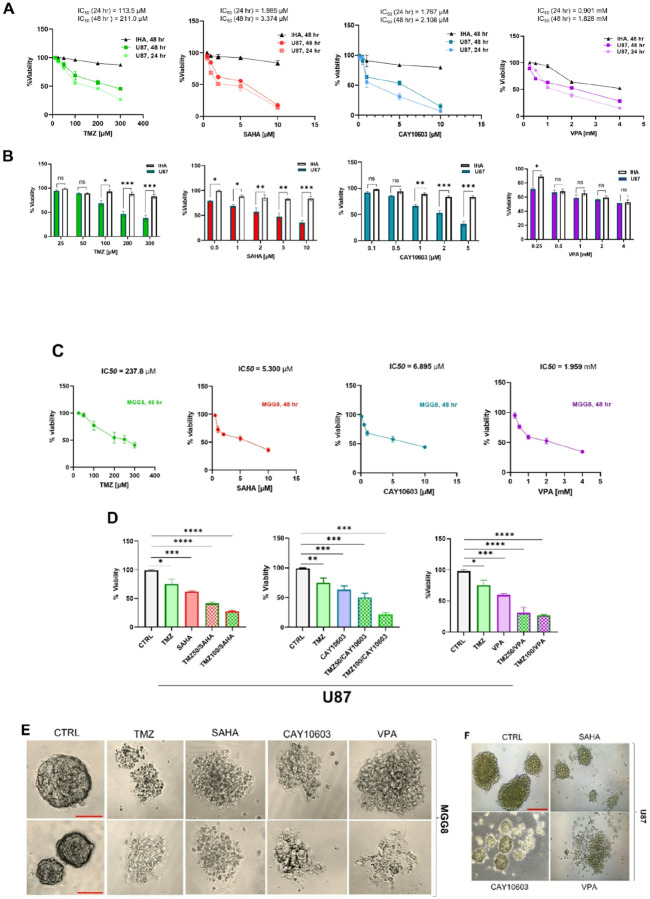
HDACis, TMZ chemotherapy, or combinatorial treatment reduce GBM cell viability in 2D and alter 3D neurosphere morphology. **A, B**. U87 and IHA cells were treated with TMZ (50–300 μM), SAHA (0.5–10 μM), CAY10603 (0.5–10 μM), and VPA (0.5–4 mM), in 2D monolayer cultures for 24 and 48 hr, compared to vehicle-treated conditions (0.01% DMSO in cell culture media). The drug effects on cell viability were assessed by MTT assay. **C**. MGG8 cells were exposed to HDACis and TMZ concentrations for 48 h, and subsequently viability was evaluated by MTT assay. Drug concentration was assessed as IC_50_. **D**. Combinatorial treatment (TMZ + HDACi, with either 50m (TMZ50) or 100 m (TMZ100) TMZ chemotherapy combined with the IC_50_ of the HDACi for 48 hours) significantly decreased U87 cell viability in 2D compared to monotherapies. **E**. Evaluation of morphological changes of MGG8 cells after 48 hr treatment with drug IC_50_ concentrations (TMZ [211 μM], SAHA [3.3 μM], CAY10603 [2.1 μM], and VPA [1.8 mM]) compared to control (CTRL) as observed via representative images of phase contrast microscopy. F. Evaluation of morphological changes of 3D GBM neurospheres (U87) after 48 hr treatment with drug IC_50_ concentrations (TMZ [211 μM], SAHA [3.3 μM], CAY10603 [2.1 μM], and VPA [1.8 mM]) compared to control (CTRL) as observed via representative images of phase contrast microscopy. * = p<0.05; ** p<0.01; ***p<0.001 vs control via Student’s t-test or multiple t-tests with Bonferroni correction (ns = not significant). Red scale bars = 500 m). Data are representative of at least three independent experiments performed in triplicate.

We evaluated the impact of HDACis on GBM cell viability of U87 and MGG8 GBM lines, as well as immortalized human astrocytes (IHAs). Cells were treated with increasing doses of VPA (0.25, 0.5, 1, 2, and 4 mM), CAY10603 (0.1, 0.5, 1, 5, and 10 μM), SAHA (0.5, 1, 2, 5, and 10 μM), and TMZ (12.5, 25, 50, 100, 200, and 300 μM) for 24 or 48 hours(Table 1). Cell viability following drug treatment was assessed using MTT assay, which revealed a dose - and time-dependent reduction in the survival of U87 cells following HDACi treatment (Fig. 1a). Importantly, the same drug concentrations showed no significant or toxic effects on IHA cells (Fig. 1b), indicating specific action of these drugs on malignant cells. At intermediate drug concentrations, differences in response between GBM cell lines were observed, with U87 (mesenchymalchemotherapeutic administration. Among the tested drug combinations, CAY10603 and TMZ exhibited the highest synergistic effect on the U87 cell line (Table 1). In 3D cultures, mimicking *in vivo* tumor growth, we observed that treatment with HDACis inhibited growth and proliferation of neurospheres, while also eliciting specific morphological changes that were drug- and cell line-dependent. After two days of treatment with an HDACi or TMZ, large MGG8 neurospheres (>500 μm in diameter) disaggregated into smaller clusters (Fig. 1e). Differentiation-associated phenotypes were observed after SAHA treatment, while CAY10603 and VPA were seen to promote dissociation of neurospheres into smaller aggregates and even single cells (Fig. 1f).

### HDACis induce apoptosis and G0/G1 and G2/M cell cycle arrest

**Figure 2:**
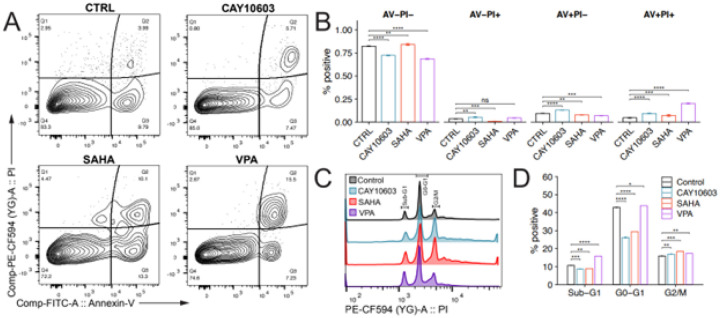
HDACis preferentially induce early/late apoptosis and cell cycle arrest in GBM cells. **A, B.** U87 cells were treated with SAHA (3.3 μM), CAY10603 (2.1 μM) and VPA (1.8 mM) for 48 h, and early and late apoptosis were detected using Annexin V/PI double staining, as analyzed by flow cytometry (early = Annexin V+/PI-; late = Annexin V+/PI+). Percent changes in the early and late apoptotic cells were calculated with respect to the untreated control, as indicated in the bar graphs. **C, D**. Cell cycle was analyzed by PI staining and flow cytometry. Sub-G1, G0/G1, S and G2/M indicate different cell cycle phases, as represented by separate peaks of PI fluorescence intensity. The bar graph shows the percentage of cells in each phase under HDACi vs untreated control U87 cells. Data are presented as mean + S.E.M. from three independent experiments with triplicates. * = p<0.05; ** p<0.01; ***p<0.001 vs control via Student’s t-tests or multiple t-tests with Bonferroni correction (ns = not significant).

To assess whether the cytotoxic effects of HDACis were associated with apoptosis, annexin V-FITC/PI double staining was performed, and apoptotic cells were quantified using flow cytometry. Treatment with SAHA and CAY10603, based on IC50 values for 48 hr, increased early apoptosis by 13.3% and 7.47%, respectively. In contrast, VPA-treated cells exhibited a significantly higher increase in late apoptosis (15.5%) compared to 10.1% and 6.71% for SAHA and CAY10603, respectively (Fig. 2a, b). These results indicate that different HDACis induce apoptosis at different rates.

We also examined the effect of HDACis on cell cycle progression. At the indicated time point (48 hours of drug treatment), treated and untreated GBM cells were collected and analyzed for cell cycle distribution using flow cytometry. In untreated U87 cells, 10.65% of cells were in the sub G1 phase. However, after 48 hours of treatment with VPA, the percentages of cells in the sub G1 phase increased to 15.8%, indicating a block at the G0/G1 phase of the cell cycle. Additionally, treatment with SAHA, CAY10603 and VPA resulted in 18.4%, 16.95% and 17.3% of cells being arrested in the G2/M phase, a slight increase compared to untreated controls (15.9%) (Fig. 2c, d). These findings suggest that HDACis induce cell cycle arrest at both the G0/G1 and G2/M phases, leading to cancer cell death and growth inhibition in vitro.

### HDACis differentially induce early versus late apoptosis in 2D/3D cultures, and modulate GBM epigenetics

To investigate the mechanisms of cell death induced by HDACis, U87 cells were treated with IC50 doses of SAHA (2.1 μM), CAY10603 (3.3 μM), and VPA (1.82 mM) for 48 hours. DAPI, PI, and caspase 3/7 localization were assessed using fluorescence microscopy. PI internalization, characteristic of late apoptotic cells was particularly evident following VPA treatment. In contrast, CAY10603 and SAHA treatments resulted in high caspase 3/7 signal intensity, confirming early apoptosis, untreated control cells showed no signs of apoptosis, as evidenced by the absence of intense red fluorescence from PI internalization (Fig. S2).

**Figure 3:**
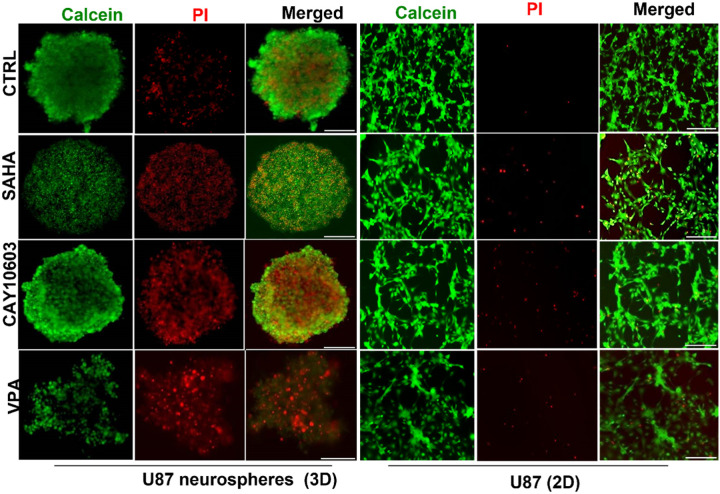
HDACis induce cell death in 3D GBM neurospheres. Confocal microscopy images show live/dead cells stained with Calcein AM (green) for viable cells and PI (red) for dead cells within 3D U87 neurospheres compared to 2D monolayers. Cell viability decreased following HDACis treatment (48 hr) compared with untreated controls. Furthermore, VPA disrupted the structure of neurospheres and increased the number of dead cells compared to SAHA and CAY10603. Scale bar = 300 μM and 20 μM for 3D and 2D cultures, respectively.

To further assess cell viability within neurospheres, dual staining with calcein-AM (for live cells) and propidium iodide (for dead cells) was performed. As shown in a representative neurosphere in Fig. 3, following SAHA treatment, most of the neurosphere consisted of dead cells, including in the central region, with few live cells remaining. In contrast, CAY10603 treatment resulted in a more even distribution of live and dead cells throughout the neurosphere. Following VPA treatment, both cell viability and neurosphere integrity were affected.

To investigate epigenetic modulation by HDACis, we next analyzed the expression of histone deacetylase 1 (HDAC1), histone deacetylase 6 (HDAC6), and histone H3 lysine 27 acetylation (H3K27ac), proteins in U87 cells treated with CAY10603, VPA, or SAHA (Fig. S1). Western blot analysis showed that VPA and SAHA treatments decreased HDAC1 expression compared to the control. Similarly, HDAC6 expression was decreased by CAY10603 and SAHA treatments. Notably, a significant increase in Cas3 expression was observed in U87 cells following treatment with SAHA and CAY10603, confirming the induction of early apoptosis by these drugs. Additionally, all three treatments —SAHA, CAY10603, and VPA—resulted in an increase in H3K27 acetylation levels compared to DMSO vehicle control.

### HDACis reduce markers for EMT, proliferation, and stemness

**Figure 4:**
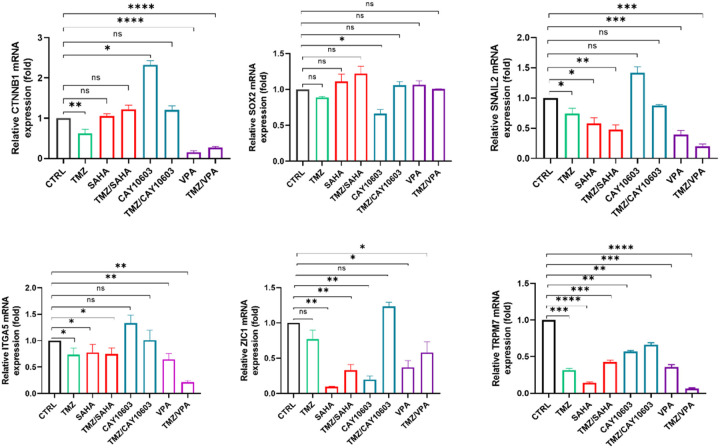
HDACi monotherapies and combinations with TMZ reduces stem-like, EMT, proliferation, and migration genes in 3D GBM neurospheres. Neurospheres derived from U87 cells were treated with SAHA, CAY10603, VPA, TMZ, combinations, or DMSO for 48 hr with IC_50_ values from Figure 1. Expression of genes related to proliferation (CTNNB1), stemness (SOX2), EMT (SNAIL2), cell-cell connections (ITGA5), and migration (TRPM7 and ZIC1) was measured by qRT-PCR, with relative gene expression reported as fold changes compared to controls. * = p<0.05; ** p<0.01; ***p<0.001 vs control via Student’s t-tests or multiple t-tests with Bonferroni correction (ns = not significant). The data are shown from three independent experiments with triplicates.

To determine if HDACis impact other tumorigenic processes by GBM cells, we assessed the expression level of key genes implicated in proliferation, stemness, apoptosis, EMT, and migration in U87 neurosphere cultures treated with HDACis and TMZ (IC_50_ values for 48 hr). PCR results showed that VPA, TMZ, and VPA/TMZ combination significantly downregulated the expression of the proliferation and migration-associated gene *CTNNB1*. TMZ, VPA, SAHA, and TMZ/SAHA also significantly downregulated the invasion-associated gene *SNAIL2*. Further assays demonstrated that CAY10603 decreased stemness and proliferation-associated gene *SOX2* expression, while CAY10603/TMZ combination therapy increased *ZIC1* expression, which suppresses cell cycle and migration (Fig. 4). Additionally, TMZ, SAHA, VPA, and TMZ/SAHA or VPA/SAHA combinational therapy decreased cell adhesion and migration-association gene *ITGA5* expression. Interestingly, all three HDACis and their individual combinations with TMZ downregulated *TRPM7* gene expression *TRPM7* is a cation channel that is overexpressed in GBM cells. It plays a role in cell growth, migration, and proliferation, and its aberrant expression is linked to GBM^21^ and as a potential drug target for GBM treatment^22,23^. Taken together, these results indicate that HDACis, even at relatively low concentrations, reduce the expression of genes associated with EMT, proliferation, and stemness.

### CAY10603 and VPA promote pro-inflammatory macrophage polarization in macrophage/GBM co-cultures

**Figure 5:**
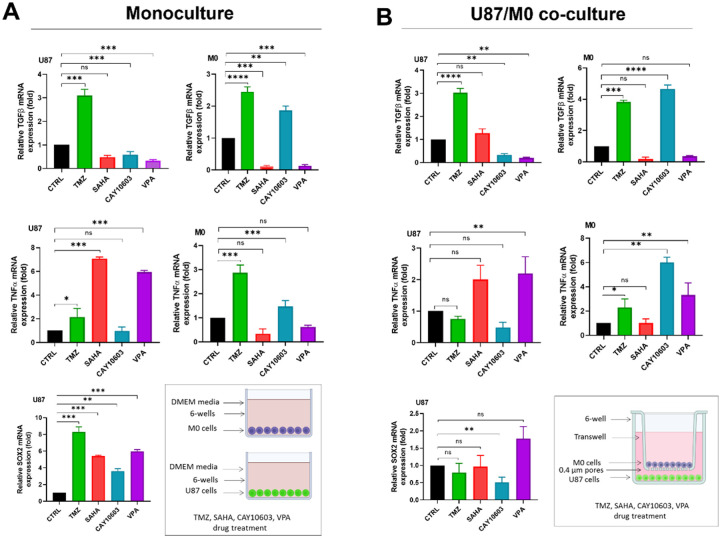
HDACis downregulate GBM stemness and anti-inflammatory genes and increase pro-inflammatory genes in GBM co-cultures with macrophages. **(A)** In monocultures of either U87 cells or M0 macrophages, generated from differentiation of human THP-1 monocytes, TGF-β expression was downregulated in THP-1 cells after SAHA and VPA treatment and in U87 cells after CAY10603 and VPA treatment. TNF-α expression was upregulated in THP-1 cells following CAY10603 and TMZ treatment and in U87 cells following VPA and SAHA treatment. **(B)** In indirect co-culture experiments of U87 and M0 macrophages in transwell assays, with U87 cells seeded on top of the transwell and macrophages seeded on the bottom, TGF-β expression was downregulated in U87 cells after CAY10603 and VPA treatment. TNF-α expression was upregulated in U87 cells after VPA treatment and in THP-1 cells following CAY10603, VPA, and TMZ treatment. SOX2 expression was downregulated in U87 cells after CAY10603 treatment, in co-culture with macrophages. Genes were evaluated by qRT-PCR, with relative gene expression and reported as fold changes compared to controls. * = p<0.05; ** p<0.01; ***p<0.001 vs control via Student’s t-tests or multiple t-tests with Bonferroni correction. (The data are shown from three independent experiments with triplicates.

Finally, because the GBM tumor microenvironment is dominated by myeloid cells^24^, we next assessed the response of human THP-1 monocytes differentiated to macrophages to TMZ and HDACis treatment. Differentiated THP-1 cells (M0) were grown either alone (monoculture) or in indirect co-culture with U87 cells for 48 hours. The expression of relevant cytokines in THP-1 cells was then analyzed by q-PCR. We found that CAY10603 and VPA simultaneously inhibited TGF-β expression in U87 cells and upregulated TNF-alpha in THP-1 macrophages, suggesting a shift towards a pro-inflammatory phenotype in the macrophages (Fig 5). Interestingly, CAY10603 was the only drug that decreased expression of the stemness marker *SOX2* in U87 co-culture. These results suggest that pharmacological inhibition of HDACs with CAY10603 and VPA reprograms macrophages toward an “M1-like” anti-tumor phenotype.

## Discussion

Numerous studies highlight the potential of HDACis in the treatment of malignant tumors, including brain cancers^14,25,26^. Notably, inhibition of class I and IIa histone deacetylases has shown antineoplastic effects in multiple preclinical studies across various solid tumors, including glioma^16,27^. Cornago et al. demonstrated that SAHA and VPA inhibit glioma cell growth *in vitro* by inducing the production of reactive oxygen species (ROS) that promote cancer cell death and alter cell cycle progression by decreasing the expression of G2 checkpoint kinases Wee1 and checkpoint kinase 1 (Chk1)^8^. Chiao et al. indicated that SAHA targets glioma stem cells through downregulation of AKT-mTOR signaling, a key suppressor of autophagy^28^. Tsai et al. demonstrated that VPA enhances the antineoplastic effect of TMZ by promoting apoptosis via activation of the p53 pathway and its downstream effector protein, PUMA^29^. Building on this literature, our study analyzed the efficacy of VPA, SAHA, and the novel small molecule CAY10603, both as monotherapies and in combination with TMZ, on GBM cell lines in 2D monolayers and 3D neurospheres, with normal astrocyte controls, and co-cultures with myeloid cells. We found that these combination treatments disturb cell cycle progression, reduce GBM cell viability, limit proliferation, and induce apoptosis. Notably, HDACis significantly enhance TMZ cytotoxic effects on GBM cells, with synergistic interaction observed between SAHA, CAY10603, VPA and TMZ in their anti-tumor activity.

SAHA (vorinostat), an FDA-approved drug for the treatment of cutaneous T-cell lymphoma^30^, is currently undergoing clinical trials for GBM, both as a monotherapy and in combination with radiotherapy^31,32^. In GBM, SAHA promotes hyper-radiosensitivity in p53 wild-type cells and cell death in GBM stem cells through activation of autophagy^33^. Similarly, VPA commonly used for seizure prophylaxis after neurosurgery, including in GBM cases^34,35^, has well-documented pharmacokinetics and toxicity evaluation in epilepsy treatment^34^. Several studies have shown that VPA sensitizes GBM cells to chemotherapy and radiotherapy by promoting apoptosis, which involves elevated p21 expression, cell cycle arrest, suppression of DNA double-strand break repair, and activation of pro-apoptotic signaling pathways^35,36^. While these pan-HDACis have been promising, recent research highlights specific overexpression of HDAC6 in GBM tissues and glioma cell lines^37,38^. Genetic knockdown of HDAC6 inhibits cell proliferation, impairs glioma stem cell activity, and sensitizes glioma cells to temozolomide (TMZ), suggesting that HDAC6 inhibitors such as CAY10603 pose as potential specific (targeted epigenetic) therapies for GBM^39,40^.

HDACis exert anti-cancer effects by modulating the tumor microenvironment and inducing apoptosis through intrinsic and/or extrinsic pathways^8,41,42^. Our cell cycle analysis revealed VPA treatment increased the apoptotic sub-G1 population. All three HDACis (SAHA, CAY10603, and VPA) also increased the G2/M phase population, suggesting an additional impact on cell growth dynamics. Notably, our study is the first to demonstrate the efficacy of CAY10603 in 2D and 3D GBM models, including induction of cell cycle arrest and promotion of apoptosis. Inhibition of cell cycle progression by HDACis—particularly at the G1/S or G2/M transition checkpoints—is a well-documented mechanism in other tumors and appears to be a critical mechanism underlying their anti-cancer activity^43,44^. Both CAY10603 and SAHA treatments also resulted in increased caspase-3 (CASP3) protein expression in U87 cells, suggesting their role in early-phase apoptosis. While, previous studies have proposed that SAHA-induced autophagy may act as a pro-survival mechanism^28,45^, our findings indicate it primarily facilitates apoptosis. In contrast, VPA primarily induced late apoptosis without altering CASP3 protein levels. These findings highlight the context-dependent effects of specific HDACis and underscore their potential as GBM therapeutic strategies.

Furthermore, earlier studies in GBM have demonstrated that VPA and SAHA suppress cell growth, induce cell cycle arrest via P21 regulation, and enhance chemotherapeutic agents^46^. Preclinical drug testing has traditionally relied on 2D *in vitro* cell models; however, these models often yield unreliable data because they fail to accurately replicate the *in vivo* tumor microenvironment. Recently, the neurosphere assay has been developed as an acceptable 3D model for maintaining GBM cells *in vitro*^47–51^. In this study, we found that HDACi treatment was associated with notable changes in neurosphere morphology. CAY10603 dramatically inhibited the formation of neurospheres (diameter ≥ 500 μm) while increasing the presence of small cell clusters (≥4 cells, diameter < 500 μm) in culture. Notably, CAY10603 was the only HDACi that additionally reduced expression of stemness markers. VPA decreased neurosphere formation, proliferation, cell-cell interactions, and epithelial-mesenchymal transition (EMT), as evidenced by the suppression of *CTNNB1*, *ITGA5*, and *SNAIL2* gene expression. Cell viability was also significantly reduced in 3D models, highlighting the potential *in vivo* translation of these agents.

Beyond the cancer cells, tumor-associated macrophages (TAMs) constitute up to 50% of the tumor bulk in glioblastoma and play an important role in tumor progression and response to chemo- and immunotherapy^52–55^. To study the effect of TAMs in GBM response to HDACis, we constructed a co-culture model consisting of differentiated THP-1 and U87 cells. We hypothesized that this co-culture would elevate immunosuppressive behavior in macrophages, which enhance stem-like phenotypes and chemoresistance in GBM cells. We found that expression of TGF-β was initially elevated in both co-cultures and monocultures, but decreased significantly following administration of VPA or SAHA in THP-1 cells, or VPA and CAY10603 in U87 cells, both in monoculture. In contrast, specific inhibition of HDAC6 by CAY10603 in THP-1/U87 co-culture both increased TNF-α expression THP-1 cells and downregulated SOX2 expression in U87 cells. We predict that HDAC6 inactivation may suppress TGF-β/SOX4 and EMT pathways through TNF-α activation. However, further research is needed to validate the proposed regulatory pathways implicated in HDACi therapy, and the epigenetically-regulated crosstalk between GBM cells and myeloid cells in the tumor microenvironment. Together, these findings underscore the importance of *in vitro* models incorporating TAMs, and suggest that CAY10603 may be effective at overcoming the immunosuppressive GBM TME and sensitizing it to immunotherapy as well as chemotherapy.

## Conclusion

The results obtained within this study demonstrate the potent and consistent anti-cancer efficacy of HDACis in GBM cells, as demonstrated by (i) sensitization to TMZ and synergistic anti-cancer effects; (ii) significant suppression of cancer stem cell phenotypes and self-renewal; (iii) marked inhibition of cell proliferation and cell cycle progression; (iv) induction of apoptosis by activation of caspase 3 and caspase 7-mediated pathways; (v) dissociation of 3D neurospheres; and (vi) therapeutically relevant induction of pro-inflammatory cytokine production in TAMs and diminished stemness properties in GBM cells through specific HDAC6 inhibition. These results suggest that SAHA, VPA, and CAY10603 – particularly as a novel small molecule inhibitor of HDAC6 – hold promise as effective combinatorial treatments for GBM, warranting further preclinical and clinical development, and highlighting the potential of HDACis in overcoming drug resistance in GBM patients.

## Supplementary Files

This is a list of supplementary files associated with this preprint. Click to download.

- SupplementaryFigure1.pdf
- SupplementaryFigure2.pdf

## Figures and Tables

**Table 1:** HDACis and chemotherapy (TMZ) dosages and their IC_50_ and CI values in GBM cells. The concentration (IC_50_) of each anticancer drug that reduced cell growth by 50% after 24 and 48 h treatments was determined from growth inhibition plots after MTT assay (Fig. 1). Data were calculated as the mean ± S.D. from three independent experiments with triplicate samples. The Combination Index (CI) was calculated for the combinations of SAHA, CAY10603, and VPA with TMZ (50 and 100 μM). Notably, the combination of TMZ (100 μM) with CAY10603 and SAHA showed the lowest CI values (i.e., most potent synergistic combinations).

Drug Concentrations Mono/combinational therapy	Cell lines	IC_50_ (24 hr.)	IC_50_ (48 hr.)	Combinational Index (CI)
TMZ 25, 50, 100, 200, 300 μM	U87 MGG8	113.55 ± 0.15	211.29 ± 0.43 237.5 ± 0.22	
SAHA 0.01, 0.1, 0.5, 1, 5, 10 μM	U87 MGG8	1.985 ± 0.29	3.374 ± 0.57 5.3 ± 0.52	
SAHA + TMZ (50 μM) SAHA + TMZ (100 μM)	U87 U87		2.158 μM 1.380 μM	0.640 0.409
CAY10603 0.01, 0.1, 0.5, 1, 5, 10 μM	U87 MGG8	1.767 ± 0.91	2.108 ± 0.89 6.895 ± 0.33	
CAY10603 + TMZ (50 μM) CAY10603 + TMZ (100 μM)	U87 U87		1.686 μM 0.4820 μM	0.799 0.228
VPA 0.25, 0.5, 1, 2, 4 mM	U87 MGG8	0.901 ±0.49	1.828 ± 0.31 1.959 ± 15	
VPA + TMZ (50 μM) VPA + TMZ (100 μM)	U87 U87		1.260 mM 1.173 mM	0.689 0.641

## Data Availability

The data that support the findings of this study are available from first author, Golnaz Asaadi Tehrani, on reasonable request.

## References

[1] Liu A., Hou C., Chen H., Zong X. & Zong P. Genetics and Epigenetics of Glioblastoma: Applications and Overall Incidence of IDH1 Mutation. Front. Oncol. 6, 16. 10.3389/fonc.2016.00016 (2016). 26858939 PMC4731485

[2] Uddin M. S. Epigenetics of glioblastoma multiforme: From molecular mechanisms to therapeutic approaches. Sem. Cancer Biol. 83, 100–120. 10.1016/j.semcancer.2020.12.015 (2022). 33370605

[3] Romani M., Pistillo M. P. & Banelli B. Epigenetic Targeting of Glioblastoma. Front. Oncol. 8, 448. 10.3389/fonc.2018.00448 (2018). 30386738 PMC6198064

[4] Hassell K. N. Histone Deacetylases and their Inhibitors in Cancer Epigenetics. Diseases 7 10.3390/diseases7040057 (2019). PMC6955926 31683808

[5] Li G., Tian Y. & Zhu W. G. The Roles of Histone Deacetylases and Their Inhibitors in Cancer Therapy. Front. Cell. Dev. Biology. 8 10.3389/fcell.2020.576946 (2020). PMC7552186 33117804

[6] Bose P., Dai Y. & Grant S. Histone deacetylase inhibitor (HDACI) mechanisms of action: emerging insights. Pharmacol. Ther. 143, 323–336. 10.1016/j.pharmthera.2014.04.004 (2014). 24769080 PMC4117710

[7] Lee J. H., Choy M. L., Ngo L., Foster S. S. & Marks P. A. Histone deacetylase inhibitor induces DNA damage, which normal but not transformed cells can repair. Proc. Natl. Acad. Sci. U S A. 107, 14639–14644. 10.1073/pnas.1008522107 (2010). 20679231 PMC2930422

[8] Cornago M. Histone deacetylase inhibitors promote glioma cell death by G2 checkpoint abrogation leading to mitotic catastrophe. Cell Death Dis. 5, e1435–e1435. 10.1038/cddis.2014.412 (2014). 25275596 PMC4237242

[9] Carew J. S., Giles F. J. & Nawrocki S. T. Histone deacetylase inhibitors: mechanisms of cell death and promise in combination cancer therapy. Cancer Lett. 269, 7–17. 10.1016/j.canlet.2008.03.037 (2008). 18462867

[10] Bezecny P. Histone deacetylase inhibitors in glioblastoma: pre-clinical and clinical experience. Med. Oncol. 31, 985. 10.1007/s12032-014-0985-5 (2014). 24838514

[11] Lo Cascio C. Quisinostat is a brain-penetrant radiosensitizer in glioblastoma. JCI Insight. 8 10.1172/jci.insight.167081 (2023). PMC10721329 37991020

[12] Mottamal M., Zheng S., Huang T. L. & Wang G. Histone deacetylase inhibitors in clinical studies as templates for new anticancer agents. Molecules 20, 3898–3941. 10.3390/molecules20033898 (2015). 25738536 PMC4372801

[13] Sanai N. Phase 0 Clinical Trial Strategies for the Neurosurgical Oncologist. Neurosurgery 85, E967–e974 (2019). 10.1093/neuros/nyz218 31245813 PMC6855937

[14] Chen R. The application of histone deacetylases inhibitors in glioblastoma. J. Exp. Clin. Cancer Res. 39, 138. 10.1186/s13046-020-01643-6 (2020). 32682428 PMC7368699

[15] Kim H. J. & Bae S. C. Histone deacetylase inhibitors: molecular mechanisms of action and clinical trials as anti-cancer drugs. Am. J. Transl Res. 3, 166–179 (2011). 21416059 PMC3056563

[16] Shi M. Q. Advances in targeting histone deacetylase for treatment of solid tumors. J. Hematol. Oncol. 17, 37. 10.1186/s13045-024-01551-8 (2024). 38822399 PMC11143662

[17] Liang T. Targeting histone deacetylases for cancer therapy: Trends and challenges. Acta Pharm. Sin B. 13, 2425–2463. 10.1016/j.apsb.2023.02.007 (2023). 37425042 PMC10326266

[18] Onwudiwe K. Single-cell mechanical assay unveils viscoelastic similarities in normal and neoplastic brain cells. Biophys. J. 123, 1098–1105. 10.1016/j.bpj.2024.03.034 (2024). 38544410 PMC11079864

[19] Seano G. Solid stress in brain tumours causes neuronal loss and neurological dysfunction and can be reversed by lithium. Nat. Biomed. Eng. 3, 230–245. 10.1038/s41551-018-0334-7 (2019). 30948807 PMC6452896

[20] Livak K. J. & Schmittgen T. D. Analysis of relative gene expression data using real-time quantitative PCR and the 2(-Delta Delta C(T)) Method. Methods 25, 402–408. 10.1006/meth.2001.1262 (2001). 11846609

[21] Wong R., Turlova E., Feng Z. P., Rutka J. T. & Sun H. S. Activation of TRPM7 by naltriben enhances migration and invasion of glioblastoma cells. Oncotarget 8, 11239–11248. 10.18632/oncotarget.14496 (2017). 28061441 PMC5355261

[22] Wong R. Inhibition of TRPM7 with waixenicin A reduces glioblastoma cellular functions. Cell. Calcium. 92, 102307. 10.1016/j.ceca.2020.102307 (2020). 33080445

[23] Chen W. L. Inhibition of TRPM7 by carvacrol suppresses glioblastoma cell proliferation, migration and invasion. Oncotarget 6, 16321–16340. 10.18632/oncotarget.3872 (2015). 25965832 PMC4599272

[24] De Leo A., Ugolini A. & Veglia F. Myeloid Cells in Glioblastoma Microenvironment. Cells 10 10.3390/cells10010018 (2020). PMC7824606 33374253

[25] Choi S. A. A novel histone deacetylase inhibitor, CKD5, has potent anti-cancer effects in glioblastoma. Oncotarget 8, 9123–9133. 10.18632/oncotarget.13265 (2017). 27852054 PMC5354719

[26] Parveen R., Harihar D. & Chatterji B. P. Recent histone deacetylase inhibitors in cancer therapy. Cancer 129, 3372–3380. 10.1002/cncr.34974 (2023). 37560925

[27] Lee P. Mechanisms and clinical significance of histone deacetylase inhibitors: epigenetic glioblastoma therapy. Anticancer Res. 35, 615–625 (2015). 25667438 PMC6052863

[28] Chiao M. T., Cheng W. Y., Yang Y. C., Shen C. C. & Ko J. L. Suberoylanilide hydroxamic acid (SAHA) causes tumor growth slowdown and triggers autophagy in glioblastoma stem cells. Autophagy 9, 1509–1526. 10.4161/auto.25664 (2013). 23962875

[29] Tsai H. C. Valproic Acid Enhanced Temozolomide-Induced Anticancer Activity in Human Glioma Through the p53-PUMA Apoptosis Pathway. Front. Oncol. 11, 722754. 10.3389/fonc.2021.722754 (2021). 34660288 PMC8518553

[30] Duvic M. & Vu J. Vorinostat: a new oral histone deacetylase inhibitor approved for cutaneous T-cell lymphoma. Expert Opin. Investig. Drugs. 16, 1111–1120. 10.1517/13543784.16.7.1111 (2007). 17594194

[31] Galanis E. Phase I/II trial of vorinostat combined with temozolomide and radiation therapy for newly diagnosed glioblastoma: results of Alliance N0874/ABTC 02. Neuro Oncol. 20, 546–556. 10.1093/neuonc/nox161 (2018). 29016887 PMC5909661

[32] Galanis E. Phase II trial of vorinostat in recurrent glioblastoma multiforme: a north central cancer treatment group study. J. Clin. Oncol. 27, 2052–2058. 10.1200/jco.2008.19.0694 (2009). 19307505 PMC2669764

[33] Diss E. Vorinostat(SAHA) Promotes Hyper-Radiosensitivity in Wild Type p53 Human Glioblastoma Cells. J. Clin. Oncol. Res. 2 (2014). PMC4219415 25379568

[34] Guo J. Valproic Acid After Neurosurgery Induces Elevated Risk of Liver Injury: A Prospective Nested Case-Control Study. Ann. Pharmacother. 56, 888–897. 10.1177/10600280211055508 (2022). 34749535

[35] Tseng J. H. Valproic acid inhibits glioblastoma multiforme cell growth via paraoxonase 2 expression. Oncotarget 8, 14666–14679. 10.18632/oncotarget.14716 (2017). 28108734 PMC5362434

[36] Thotala D. Valproic acid enhances the efficacy of radiation therapy by protecting normal hippocampal neurons and sensitizing malignant glioblastoma cells. Oncotarget 6, 35004–35022. 10.18632/oncotarget.5253 (2015). 26413814 PMC4741505

[37] Auzmendi-Iriarte J. Characterization of a new small-molecule inhibitor of HDAC6 in glioblastoma. Cell Death Dis. 11, 417. 10.1038/s41419-020-2586-x (2020). 32488056 PMC7265429

[38] Garrett M. HDAC1 and HDAC6 are essential for driving growth in IDH1 mutant glioma. Sci. Rep. 13 10.1038/s41598-023-33889-3 (2023). PMC10394035 37528157

[39] Wang Z. HDAC6 promotes cell proliferation and confers resistance to temozolomide in glioblastoma. Cancer Lett. 379, 134–142. 10.1016/j.canlet.2016.06.001 (2016). 27267806

[40] Spallotta F. & Illi B. The Role of HDAC6 in Glioblastoma Multiforme: A New Avenue to Therapeutic Interventions? Biomedicines 12 (2024). 10.3390/biomedicines12112631 PMC11591585 39595195

[41] Moran B., Davern M., Reynolds J. V., Donlon N. E. & Lysaght J. The impact of histone deacetylase inhibitors on immune cells and implications for cancer therapy. Cancer Lett. 559, 216121. 10.1016/j.canlet.2023.216121 (2023). 36893893

[42] Li Y. & Seto E. HDACs and HDAC Inhibitors in Cancer Development and Therapy. Cold Spring Harb Perspect. Med. 6 10.1101/cshperspect.a026831 (2016). PMC5046688 27599530

[43] Hoffmann M. J., Meneceur S., Hommel K., Schulz W. A. & Niegisch G. Downregulation of Cell Cycle and Checkpoint Genes by Class I HDAC Inhibitors Limits Synergism with G2/M Checkpoint Inhibitor MK-1775 in Bladder Cancer Cells. Genes (Basel). 12. 10.3390/genes12020260 (2021). PMC7916885 33670166

[44] Takai N. Histone deacetylase inhibitors have a profound antigrowth activity in endometrial cancer cells. Clin. Cancer Res. 10, 1141–1149. 10.1158/1078-0432.ccr-03-0100 (2004). 14871994

[45] Mrakovcic M., Kleinheinz J. & Fröhlich L. F. Histone Deacetylase Inhibitor-Induced Autophagy in Tumor Cells: Implications for p53. Int. J. Mol. Sci. 18 10.3390/ijms18091883 (2017). PMC5618532 30563957

[46] Zhao L. Histone deacetylation inhibition in pulmonary hypertension: therapeutic potential of valproic acid and suberoylanilide hydroxamic acid. Circulation 126, 455–467. 10.1161/circulationaha.112.103176 (2012). 22711276 PMC3799888

[47] Quereda V. A Cytotoxic Three-Dimensional-Spheroid, High-Throughput Assay Using Patient-Derived Glioma Stem Cells. SLAS Discov. 23, 842–849. 10.1177/2472555218775055 (2018). 29750582 PMC6102052

[48] Kogiso M. Concurrent Inhibition of Neurosphere and Monolayer Cells of Pediatric Glioblastoma by Aurora A Inhibitor MLN8237 Predicted Survival Extension in PDOX Models. Clin. Cancer Res. 24, 2159–2170. 10.1158/1078-0432.Ccr-17-2256 (2018). 29463553 PMC6052861

[49] Musah-Eroje A. & Watson S. A novel 3D in vitro model of glioblastoma reveals resistance to temozolomide which was potentiated by hypoxia. J. Neurooncol. 142, 231–240. 10.1007/s11060-019-03107-0 (2019). 30694423 PMC6449313

[50] Kloepper J. Ang-2/VEGF bispecific antibody reprograms macrophages and resident microglia to anti-tumor phenotype and prolongs glioblastoma survival. Proceedings of the National Academy of Sciences 113, 4476–4481 (2016). 10.1073/pnas.1525360113 PMC4843473 27044098

[51] Peterson T. E. Dual inhibition of Ang-2 and VEGF receptors normalizes tumor vasculature and prolongs survival in glioblastoma by altering macrophages. Proceedings of the National Academy of Sciences 113, 4470–4475 (2016). 10.1073/pnas.1525349113 PMC4843449 27044097

[52] Khan F. Macrophages and microglia in glioblastoma: heterogeneity, plasticity, and therapy. J. Clin. Invest. 133 10.1172/jci163446 (2023). PMC9797335 36594466

[53] Landry A. P., Balas M., Alli S., Spears J. & Zador Z. Distinct regional ontogeny and activation of tumor associated macrophages in human glioblastoma. Sci. Rep. 10, 19542. 10.1038/s41598-020-76657-3 (2020). 33177572 PMC7658345

[54] Datta M. Losartan controls immune checkpoint blocker-induced edema and improves survival in glioblastoma mouse models. Proceedings of the National Academy of Sciences 120, e2219199120 (2023). 10.1073/pnas.2219199120 PMC9963691 36724255

[55] Burchett A., Siri S., Li J., Lu X. & Datta M. Novel 3-D Macrophage Spheroid Model Reveals Reciprocal Regulation of Immunomechanical Stress and Mechano-Immunological Response. Cell. Mol. Bioeng. 17, 329–344. 10.1007/s12195-024-00824-z (2024). 39513012 PMC11538219

